# Theranostic Strategy of Focused Ultrasound Induced Blood-Brain Barrier Opening for CNS Disease Treatment

**DOI:** 10.3389/fphar.2019.00086

**Published:** 2019-02-07

**Authors:** Ko-Ting Chen, Kuo-Chen Wei, Hao-Li Liu

**Affiliations:** ^1^Ph.D. Program in Biomedical Engineering, Chang Gung University, Taoyuan, Taiwan; ^2^Department of Neurosurgery, Chang Gung Memorial Hospital at Linkou, Taoyuan, Taiwan; ^3^Department of Electrical Engineering, Chang-Gung University, Taoyuan, Taiwan

**Keywords:** focused ultrasound, blood-brain barrier, brain drug delivery, brain tumor, Alzheimer's disease

## Abstract

Focused Ultrasound (FUS) in combination with gaseous microbubbles has emerged as a potential new means of effective drug delivery to the brain. Recent research has shown that, under burst-type energy exposure with the presence of microbubbles, this modality can transiently permeate the blood-brain barrier (BBB). The bioavailability of therapeutic agents is site-specifically augmented only in the zone where the FUS energy is targeted. The non-invasiveness of this approach makes FUS-induced BBB opening a novel and attractive means to perform localized CNS therapeutic agent delivery. Over the past decade, FUS-BBB opening has been preclinically confirmed to successfully enhance CNS penetration of therapeutic agents including chemotherapeutic agents, therapeutic peptides, monoclonal antibodies, and nanoparticles. Recently, a number of clinical human trials have begun to explore clinical utility. This review article, explores this technology through its physical mechanisms, summarizes the existing preclinical findings (including current medical device designs and technical approaches), and summarizes current ongoing clinical trials.

## Mechanism

### The Blood-Brain Barrier (BBB)

The blood-brain barrier (BBB) is the major part of the brain's neurovascular unit (NVU) and serves as a key homeostatic site for the central nervous system (CNS), maintaining both structural, and functional brain connectivity (Zhao et al., 2015). The BBB is composed of specialized highly polarized endothelial cells, pericytes, and astrocytic processes and develops through a multi-step process starting in the neuro-ectoderm with angiogenesis followed by endothelium growth (Zhao et al., 2015; Maiuolo et al., 2018; Warren, 2018). The capillary endothelium composes of the majority of the BBB surface area (>85%) and numerous transport systems facilitate or actively shuttle molecules across the BBB (Sweeney et al., 2018). Dysfunction of BBB permeability and transporters lead to various kinds of neurological disorders, including stroke, Alzheimer's, Huntington's, Parkinson's, amyotrophic lateral sclerosis, multiple sclerosis, various types of infectious disease and even neoplasms, which may alter the regional or even global cerebral microenvironment (Schoknecht et al., 2015; Nelson et al., 2016; Maiuolo et al., 2018; Sweeney et al., 2018). Therapeutic targets have been proposed to treat a broad spectrum of disease, but must first cross the BBB for effective drug delivery or to increase waste elimination (e.g., amyloid **β**) (Nelson et al., 2016; Sweeney et al., 2018).

### Various Approach to Overcome BBB

Many drug molecules and therapeutics cannot naturally permeate the BBB into the brain parenchyma, presenting a serious challenge to treating brain disorders. Several methods of penetrating the BBB can be categorized as physical or non-physical. In physical delivery methods, an opening of tight junctions between endothelial cell barriers provides paths by which molecules can diffuse passively into the brain parenchyma. Osmotic agents, offering globally transient disruption of the BBB via osmotic shrinkage of endothelial cells and through creating an osmotic pressure gradient across the BBB, are widely used for drug delivery for brain tumor patients (Rodriguez et al., 2015). Concurrent intra-arterial administration of osmotic and chemotherapeutic agents has raised patient survival from 11.4 to 17.5 months (Gumerlock et al., 1992). Nevertheless, due to its systemic effect rather than localized BBB alterations, complications such as neurological deficits, seizures and potential tumor migration have been reported (Gumerlock et al., 1992). On the other hand, invasive procedures such as direct injection can specifically target the brain compartment and cells of interest, removing the loss of first pass clearance and off-target toxicity (Duskey et al., 2017). Chemotherapeutic agents can be delivered interstitially by local injection or drug-carrying biodegradable matrices can be directly implanted into the debulked tumor cavity (Westphal et al., 2003). Convection-enhanced delivery (CED) interstitially infuses drugs under a constant pressure gradient, producing bulk interstitial fluid flow through the brain following the opening of the skull (Ferguson et al., 2007). Animal models show CED achieves greater localized penetration of chemotherapy drugs than intravenous administration, but the local distribution is dependent on the volume and rate of the gradient of infusion, along with the drug's concentration, polarity and molecular weight. Low infusion rates and volumes can result in highly inconsistent distribution and tumor interstitial fluid pressure, resulting in rapid efflux of drugs from the injection site. In addition, the insertion of objects into the brain is inherently invasive, and can increase the likelihood of infection or damage.

In non-physical delivery, drug molecules and therapeutics are systemically delivered to the luminal side of the BBB. The design of such approaches must consider several hurdles including first pass clearance, blood instability, immune response, and off-target effects (Chen et al., 2010; Upadhyay, 2014; Duskey et al., 2017). Viral- or nanopartical-based modification of therapeutics seek to penetrate the BBB through active or passive crossing non-specific or receptor-mediated uptake (Duskey et al., 2017). A more effective solution may involve combining both physical and non-physical methods, such as combining physical methods with nanoparticles or advanced bioconjugate technologies to enhance the delivery while simultaneously stabilizing proteins or enzymes as necessary (Duskey et al., 2017).

### FUS-Induced BBB Opening Concepts

**Figure 1:**
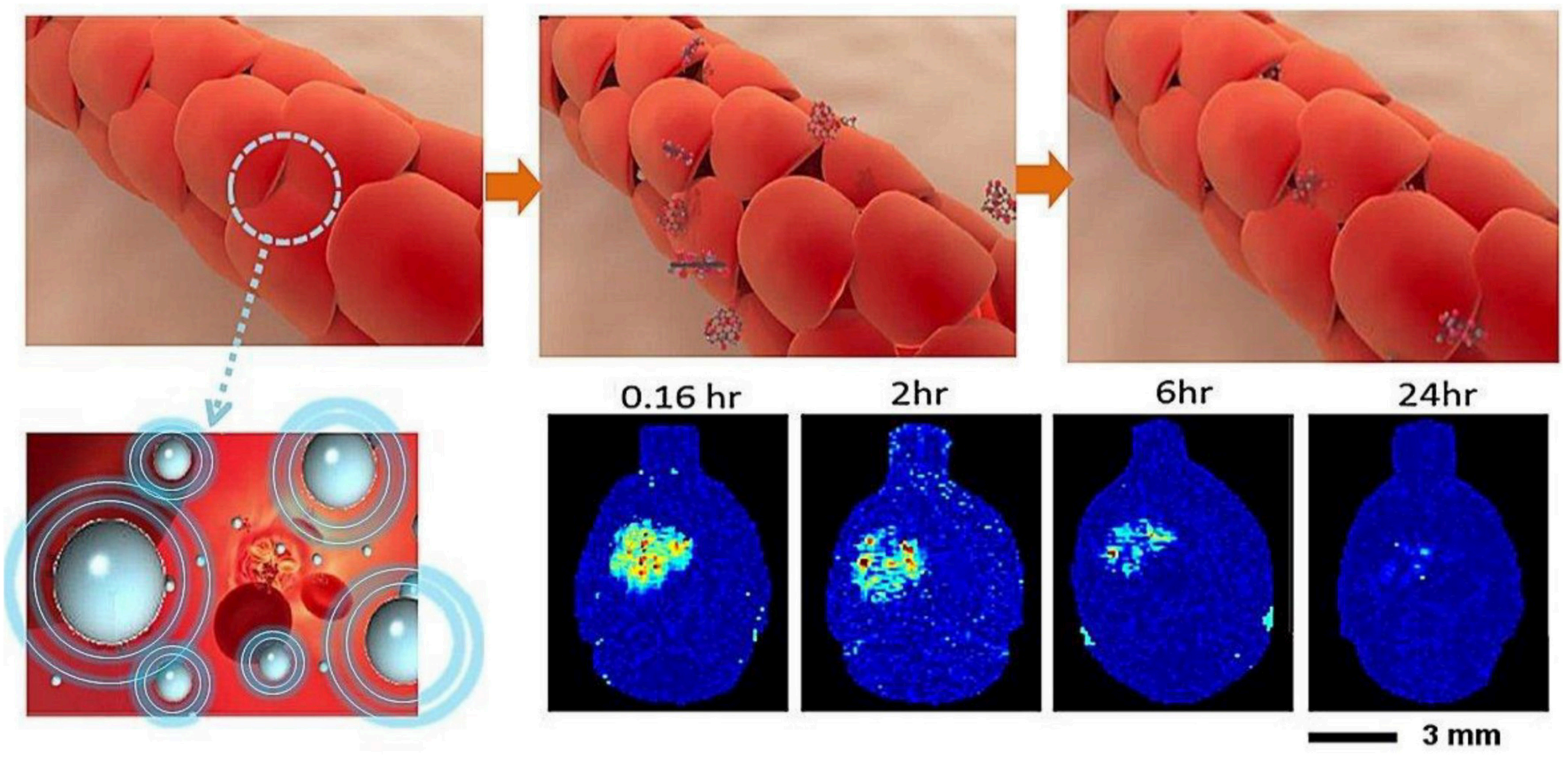
BBB opening concepts: interaction of microbubbles and focused ultrasound transiently disrupts the tight junction of the capillary lumen to allow therapeutic agents to penetrate into the brain. The BBB return to normal a few hours following focused ultrasound exposure (Chai et al., 2014).

The BBB blocks nearly 98% of drug compounds from accessing the CNS, and the use of focused ultrasound raises the potential for developing a drug delivery platform (Pardridge, 2005). The permeability of the BBB can be transiently increased using low-energy burst-tone focused ultrasound following an administration of intravenous microbubbles (Hynynen et al., 2001, 2003; Park et al., 2012; Chai et al., 2014). A physical cavitation effect is created from circulating microbubbles, significantly reducing the ultrasound pressure to produce an equivalent acoustic cavitation effect (concepts see Figure 1). The subsequent application of ultrasonic energy can achieve a local detachment of tightly sealed junctions on the capillary wall without inducing neuronal damage (Hynynen et al., 2005). Due to its spherically concaved transducer design, ultrasonic energy focused at the geometrical center can be sharply cascaded, allowing ultrasonic energy to be tightly deposited deeply within the brain tissue while minimizing skull energy absorption (Clement et al., 2000). Since the BBB blocks nearly 98% of drugs from accessing the CNS, the use of focused ultrasound raises a potential therapeutic delivery platform to the CNS (Pardridge, 2005).

Focused ultrasound could focally and transiently open the BBB to introduce drugs via a physical delivery, which has several advantages. Compared with alternative routes, such as using osmotic agents or modified lipophilic chemicals via intravascular infusion, FUS can locally increase BBB permeability (Doolittle et al., 2000; Pardridge, 2002). Compared with other physical approach like CED, FUS is a less invasive method. While non-physical delivery methods use a different mechanism, which depends on cellular non-specific or receptor uptake, to overcome BBB, FUS holds abovementioned advantages and provides high flexibility in combing with various CNS treatment modalities (Hsu et al., 2013; Fan et al., 2016).

## Preclinical Technical Validation

### Biophysical Observation Caused by FUS-BBB Opening

Several tight junction integrated adhesion molecules, including claudin-1, claudin-5, and ZO-1, can be transiently regulated by FUS (Sheikov et al., 2006, 2008). Glial fibrillary acidic protein staining confirms that FUS-BBB opening triggers the activation of microglia and astrocytes (Alonso et al., 2011). It has been reported that ultrasound can temporarily suppress P-glycoprotein expression, the most dominant multi-drug resistant protein found in the BBB, for days even after BBB closure (Cho et al., 2016). FUS-BBB opening may trigger acute transcriptional changes, particularly a transient inflammatory response in microvessels, but the increased transcription of proinflammatory cytokine genes appears to quickly return to the baseline (Kovacs et al., 2017; McMahon et al., 2017).

### Modality to Identify BBB Opening

**Figure 2:**
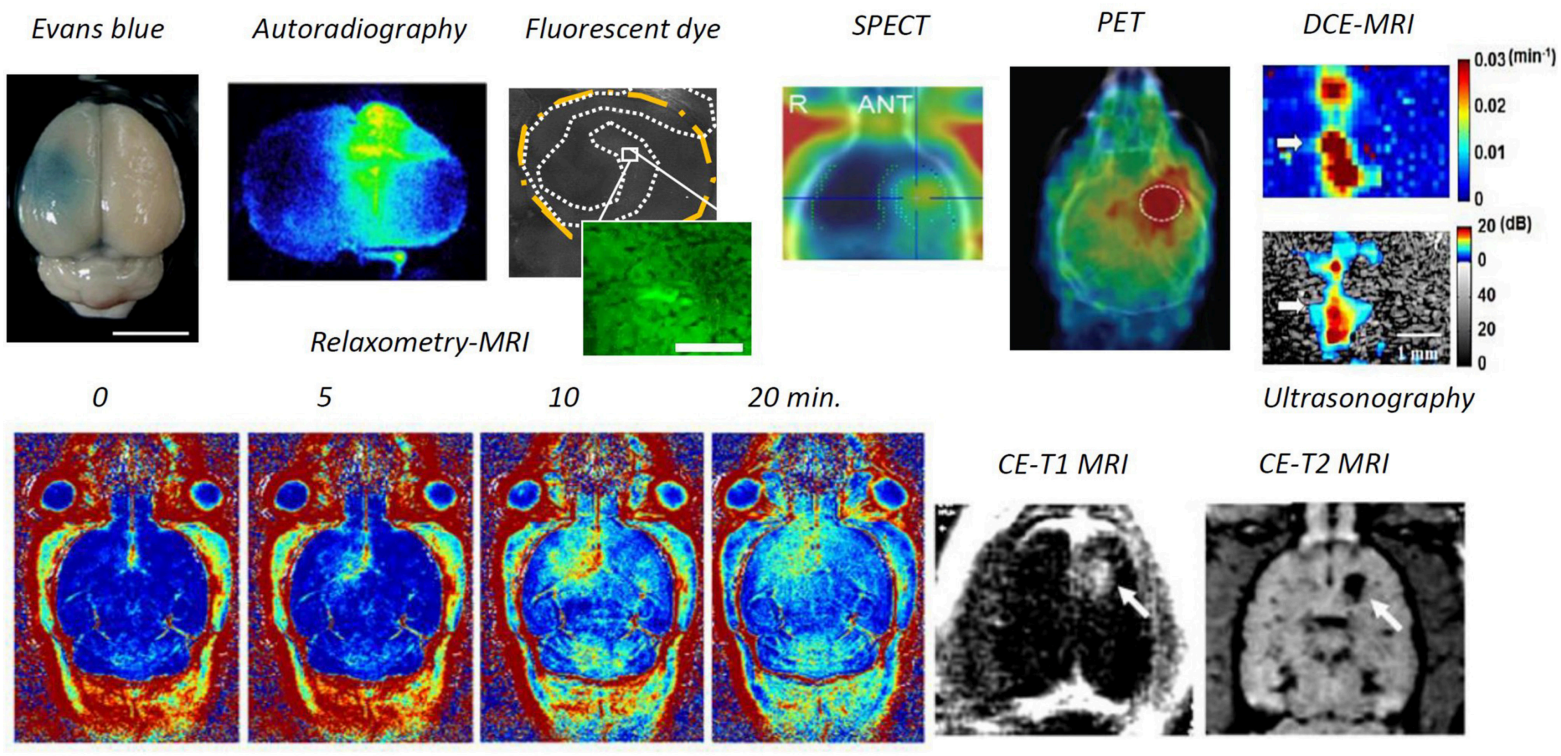
Modalities to identify BBB opening. Through *ex-vivo* examination, Evans blue dye can directly depict the BBB-opened region from gross section, or fluorescent dextran or the radioactivity readout through autoradiography from the brain gross section can be used to identify the BBB-opened region. Previous attempts have included *in-vivo* examination, ultrasonography via microbubble dynamic characterization, SPECT/ PET via radiotracer, contrast-enhanced MRI either via Gd-DTPA or MNPs), and dynamic contrast-enhanced MRI via Gd-DTPA (Lin et al., 2009; Liu et al., 2009, 2010a, 2016; Chai et al., 2014; Fan et al., 2014; Xia et al., 2016; Wu et al., 2017).

Numerous tools have been developed to identify BBB opening (see Figure 2). Direct microscopic observations of BBB-opened phenomena have been made at the cellular scale. An *in-vivo* imaging approach was designed to monitor the pharmacodynamic behavior of BBB-opening. By providing an indicator of diethylenetriamine penta-acetic acid (Gd-DTPA; molecular size about 1 kDa), dynamic contrast enhanced magnetic resonance imaging (DCE-MRI) can be used to monitor the kinetic behavior of the T1-weithed MRI contrast agent, thus the transient BBB opening is estimated to have a half-life of 2–5 h based on the acoustic pressure level (Park et al., 2012; Chai et al., 2014). Compare with quantification through a surrogate molecule (Evans blue), a strong association was found between kinetic behavior and the 70-kDa surrogate, thus imaging contrast agents could be used as a molecule-delivered surrogate (Chai et al., 2014).

In addition to contrast-enhanced T1-weighted MRI, various other imaging tracers have been delivered across the BBB, including horseradish peroxidase (Hynynen et al., 2005), lanthanum chloride (Sheikov et al., 2008), and ionic manganese (Howles et al., 2010) from immunohistochemistry based microscopy; Alexa Fluor 488 (Raymond et al., 2007), Texas-Red-tagged dextran (Choi et al., 2010) and GFP-tagged dextran (Liu et al., 2016) from fluorescent microscopy; 99 mTc diethylenetriamine pentaacetate and 68-Ga-surrogate compound through nuclear imaging SPECT/ PET (Lin et al., 2009; Liu et al., 2016); superparamagnetic iron oxide (SPIO, 60 nm) through T2-weighted MRI (Liu et al., 2009); and gold nanorods through photoacoustic imaging (Wang et al., 2012).

### Physical Characterization

#### BBB Opening Associated With Acoustic Cavitation

Inertial and stable microbubble-present acoustic cavitation can be characterized from distinct backscattered acoustic emissions (McDannold et al., 2006). Acoustic cavitation is a physical effect produced by gas-filled bubbles after exposure to certain ultrasound frequencies, causing harmonic microbubble compression and expansion (Crum et al., 1992; Stride and Saffari, 2003). Acoustic cavitation contributes to BBB-opening through stable or inertial cavitation. Stable cavitation directly contributes to tight junctional disruption (McDannold et al., 2006), while inertial caviation can result in additional erythrocyte extravasations (Liu et al., 2008).

In stable cavitation, ultrasound stimulation causes repetitive microbubble volumetric oscillation. The expansion of the microbubbles separates the endothelial cell lining, and contraction causes invagination of the vascular lining. This push-pull action broadens tight junctions in the BBB (Caskey et al., 2007). Rapid oscillation of microbubbles also results in consistent microstreaming, which can stimulate the capillary endothelium, thus increasing shear stress on cells, damaging the endothelial lining and enhancing internal cell permeability (Sboros, 2008). Excessive ultrasound energy results in the sudden collapse of microbubbles (i.e., inertial cavitation), producing strong mechanical stress, microstreaming, and micro-jets in the surrounding media (Husseini et al., 2005), inducing cellular membrane perforation and large-scale blood-tissue permeation (Mitragotri, 2005), along with erythrocyte extravasations or micro-hemorrhages (Hynynen et al., 2005; Liu et al., 2008). Inertial cavitation is characterized by a wideband emission causing microbubble collapse and disruption, and a stable cavitation is characterized by subharmonic/ultraharmonic emissions which produce a stable contraction and expansion of microbubbles (Bader and Holland, 2013; Jin et al., 2016).

Clinical applications of FUS-BBB opening require the development of indices to assess the likelihood of such opening occurring, to allow for the assessment and estiation of CNS therapeutic molecule delivery. Passive cavitation dose (PCD) analysis is applied to microbubble activity to detect and characterize backscattered acoustic emissions. FUS-induced BBB opening is both associated with inertial cavitation and likely caused by stable cavitation (O'Reilly and Hynynen, 2012; Chen and Konofagou, 2014; Marquet et al., 2014; Sun et al., 2015). A mechanical index (MI) is defined as the peak negative acoustic pressure over the square root of the frequency (i.e., MI = P/√ f, P in MPa, f in MHz) and is used to assess ultrasound-induced mechanical bio-effects (Apfel and Holland, 1991). McDannold et al. identified a strong association between the degree of FUS-induced BBB opening and MI using signal intensity (SI) change to contrast-enhanced magnetic resonance imaging (CE-MRI), identifying a threshold which serves as an indicator for BBB opening (McDannold et al., 2006). Despite reports of this correlation, the level of MI is usually seen as a reflection of the extent of inertial cavitation (Apfel and Holland, 1991). The cavitation index (CI) also serves as an indicator of stable microbubble-ultrasound cavitation. Bader et al. used the CI, defined as peak negative acoustic pressure (in MPa) over frequency (in MHz); i.e., CI = P/f, to assess the chance of subharmonic emissions being caused by stable microbubble-presented cavitation activity (Bader and Holland, 2013), which is highly associated with the extent of BBB opening (McDannold et al., 2007). We recently used dynamic contrast-enhanced (DCE)-MRI and PCD analysis to assess the feasibility of gauging the extent of FUS-induced BBB opening using MI and CI,. DCE-MRI was found to evaluate pharmacodynamics/pharmacokinetic BBB-opening dynamics, and was strongly associated with both with MI and CI, implying the feasibility in using these two indices to gauge the scale of FUS-induced BBB opening (Chu et al., 2016).

#### Inference of Ultrasound Exposure on BBB Opening

Several preclinical studies have used a range of FUS parameters for FUS-induced BBB opening, including exposure frequency, acoustic pressure, burst length, pulse-repetition frequency, and duration (Hynynen et al., 2005; McDannold et al., 2008; O'Reilly et al., 2011). The acoustic pressure of sonication (i.e., different types of cavitation) can modulate the leakage kinetics of fluorescent dye in the cerebral vasculature, and can be used to characterize leakage as fast or slow (Cho et al., 2011; Nhan et al., 2013). During high-pressure exposure, the oscillating microbubbles cause a direct and immediate broadening of tight junctions and pores on the cell membrane, but low-pressure exposure causes microbubble oscillation to activate endothelial cell receptors, thus promoting the trans-cellular transport of molecules from the lumen side to the abluminal space (Deng et al., 2012).

#### Microbubble

Microbubbles (MB) assume an important part in the FUS-induced BBB opening effect. Currently, commercialized MBs include Optison (GE Healthcare, WI, USA), Definity® (Lantheus Medical Imaging, MA, USA), and SonoVue® (Bracco, Milano, Italy). All have received FDA approval for diagnostic use and have been used for FUS-induced BBB opening. Commercial MBs generally exceed 2 μm in diameter and have an application window of 5–10 min. Each, however have different compositions, concentrations, half-lives, and hydrodynamic sizes, which must be considered in terms of impact on interaction between ultrasound-MBs and capillary permeability. McDannold et al. achieved BBB-opening using similar acoustic pressure thresholds for Optison™ (human serum albumin) and Definity® (lipid) MBs (McDannold et al., 2007), though Optison™ produced a more serious bio-effect, possibly because the lipid shell in Definity® is stronger than the albumin shell in Optison™. We assessed the impact of BBB opening using three different MBs–SonoVue®, Definity®, and USphere® in combination with FUS. Under identical MB concentrations, all induced similar and equivalent BBB-opening effects (Wu et al., 2017).

In addition to MB type, the concentration of injected MBs produces various numbers of nuclei for cavitation within the vasculature, which can also significantly affect the distribution and degree of BBB opening (McDannold et al., 2008; Yang et al., 2008). An increase of MB volume would increase the mechanical force acting on nearby cells, thus expanding to a size sufficient to stimulate the vessel walls. Previous studies have found that, compared with larger Mbs (4–5 μm and 6–8 μm), MBs with a diameter of 1–2 μm offer significantly less permeability enhancement (Vlachos et al., 2011). Meanwhile, smaller (1–2 μm) MBs have been reported to reduce recovery time following transient BBB opening (Samiotaki et al., 2012). Considering the effect of total MB volume on BBB opening, Song et al. have demonstrated that, to optimize BBB-opening efficiency, size and concentration can be merged into one single parameter, microbubbles gas volume dose (Song et al., 2017). The duration of the BBB-opening effect has been found to depend on the degradation dynamics of each MB type. Wu et al. delivered a large treatment volume through multiple exposures, thus compensating for MB degradation and producing a more durable BBB-opening effect (Wu et al., 2017).

### Intraoperative Monitoring and Guidance

**Figure 3:**
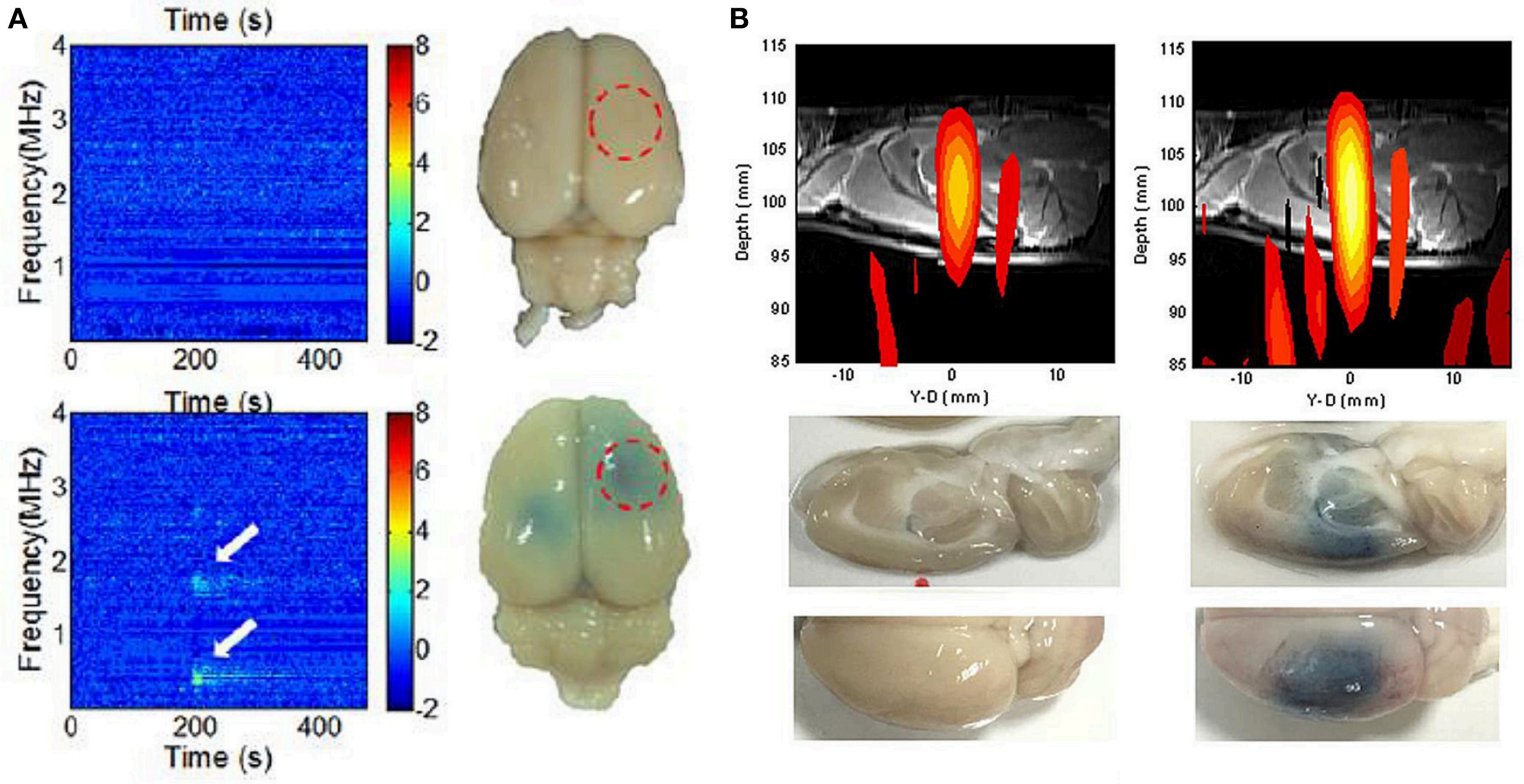
Intraoperative monitoring and guidance of focused ultrasound-induced BBB opening. **(A)** Detection of subharmonic/ ultraharmonic of backscattered spectrum for real-time BBB opening monitoring (Tsai et al., 2016). **(B)** Dual transmit/receive mode ultrasound phase array to intraoperatively reconstruct focal beam for ultrasound energy guidance (Liu et al., 2018).

Although FUS-BBB opening appears promising, FUS energy must be precisely controlled to avoid adverse effects including massive erythrocyte extravasation (Hynynen et al., 2005; Liu et al., 2008). Indeed, DCE-MRI can indicate BBB-opening by postoperatively detecting contrast medium leakage into the brain parenchyma, a real-time monitoring scheme is required to provide instant intraoperative feedback to assure the safety and effectiveness of FUS energy exposure (Hynynen et al., 2005). To integrate diagnostic ultrasound into the therapeutic transducer to passively receive the backscattered emission waves provides a potential approach for intraoperative beam mapping and monitoring. Passive cavitation detection (PCD), therefore, served as a tool for real-time transcranial monitoring during FUS, and served as an online treatment evaluation complement to the postoperative MRI-based methods (see Figure 3) (Wu et al., 2016). The passive image is reconstructed using passive beam formation theory originally developed for seismic source identification (Jensen et al., 2012; Arvanitis et al., 2013). Ultrasound research has recently focused on passive imaging as a way to monitor bubble activity during cavitation-enhanced therapy, thus improving safety and outcome assessments (Lin et al., 2009; Liu et al., 2009, 2016; Choi et al., 2010; Howles et al., 2010). Good synchronization between therapeutic exposure and diagnostic backscattered reception allows for focal beam reconstruction at the sub-MPa level without the use of MBs (Xia et al., 2016; Liu et al., 2018). To improve transcranial detectability, an alternative to a separate transmission/receiving transducer is to match the backscatter reception with the transmission ultrasound frequency. O'Reilly et al. proposed a lateral-mode vibration large-scale hemispherical phased array structure, with a low-density PVDF membrane covering the hemisphere to locate the microbubble activation source (O'Reilly et al., 2014), producing a high-resolution tracking of the MB distribution that can be used for real-time monitoring of the BBB-opening process.

Other researchers have sought to use passive acoustic detection to detect cavitation activity and successfully predict FUS-BBB opening. McDannold et al. used multiple piezoelectric elements to receive emissions during FUS exposure, and confirmed that FUS-BBB opening can occur without wideband emission (McDannold et al., 2006). They also found good correlation with receiving passive signals, with BBB-opened sites providing increased second and third harmonic signal levels (McDannold et al., 2012). Tung et al. achieved FUS-BBB opening without inertial cavitation, and proposed that a higher order (i.e., fourth or fifth) harmonic level change is associated with FUS-BBB opening (Deng et al., 2012; Nhan et al., 2013). Vykhodtseva et al. detected subharmonic emissions during FUS exposure (Vykhodtseva et al., 1995). O'Reilly and Hynynen detected ultraharmonic components using a wideband polyvinylidene-difluoride (PVDF) receiver as an indicator of BBB-opening detection, and showed high detectability and success rate for BBB-opening (O'Reilly and Hynynen, 2010). Subsequently, more recent attempts have sought to use acoustic emission detection technologies (particularly harmonic and ultraharmonic) for real-time tracking of FUS-BBB opening. Arvanitis et al used a PVDF hydrophone to track changes in the magnitude of an integrated component set (2×, 3×, and 4× harmonics and 1.5× and 2.5× ultraharmonics) to detect FUS-BBB opening (Arvanitis et al., 2012). Sun et al. used passive cavitation activity detection to monitor BBB-opening (Sun et al., 2015). Other research also indicates that subharmonics or ultraharmonics correlate better with BBB opening (O'Reilly and Hynynen, 2010, 2012; Arvanitis et al., 2012). A dual-confocal transducer was also used to improve subharmonic PCD for highly accurate prediction of BBB-opening, raising the potential for application in real-time ultrasound BBB opening control (Tsai et al., 2016).

### Brain Tumor Treatment

#### Clinically Approved Therapeutic Agent Delivery

The BBB shows heterogeneous integrity within tumor tissues, meaning the degree of permeability can vary within a single tumor. The core region of a tumor is usually more permeable than the periphery (Ewing et al., 2006). In gliomas, the integrity of the BBB in peripheral areas has been shown to remain highly functional (Groothuis et al., 1982; Neuwelt et al., 1982). In treating an intrinsic brain tumor, such as a glioma, the intact BBB of the tumor periphery limits drug penetration and treatment success. Effectively enhancing BBB permeability of the tumor periphery represents a potential strategy for improving treatment efficacy and ultimately patient survival.

Enhanced drug delivery via MB-assisted FUS-BBB opening is widely established. Herceptin (150 kDa) and D4 receptor antibodies (150 kDa) have been successfully delivered into mouse brain (Kinoshita et al., 2006). Methotrexate (545 Da) has also been delivered into normal rat brains in the FUS-assisted model at significantly higher concentrations than in control rats (Mei et al., 2009). The earliest attempt for FUS-enhanced drug delivery for glioma treatment used doxorubicin as Doxil® (Ben Venue Laboratories, OH, USA) encapsulated in the form of long-circulating pegylated liposomes (Treat et al., 2007, 2012). Enhanced delivery of boronophenylalanine, with a high thermal neutron capture cross-section for boron neutron-capture therapy (BNCT), has been achieved via MB-FUS BBB opening, indicating that this technique has potential for increasing the treatment efficiency of BNCT (Yang et al., 2012; Alkins et al., 2013). Another drug called BCNU, which has been used for many years as a chemotherapeutic agent for treating glioma patients, was also tested in an MB-FUS-enhanced model. Although BCNU is lipophilic, meaning it has potential to penetrate BBB, its substantial toxicity limits the overall dosage and thus concentrations in tumors (Liu et al., 2010a). We also demonstrated enhanced TMZ delivery by MB-FUS BBB opening. A liquid chromatography–tandem mass spectrometer was used to measure the TMZ levels in both CSF and plasma (Wei et al., 2013a; Liu et al., 2014). Finally, the enhanced delivery of an antiangiogenic monoclonal antibody, bevacizumab, has been shown to significantly retard glioma progression, leading to a markedly increased median survival in animal models (Liu et al., 2016).

#### Novel Multi-functioned Therapeutic Agent Design for Glioma Treatment

Besides using microbubbles as a catalyst to induce BBB opening, the MB itself has been designed as a carrier of therapeutic drugs. Encapsulating therapeutic agents in or conjugating them with MBs is a more recent approach. Therapeutic agents have been incorporated into in MB carriers by attachment to the outer shell surface, embedding in the shell, dissolving hydrophobic drugs in the oily layer between the gas core and shell, and by linking them to the shell (Unger et al., 2004; Hernot and Klibanov, 2008). Drugs can also be pre-incorporated into carriers such as liposomes, micelles, or microspheres which can be easily attached to lipid MBs, usually via avidin–biotin interactions (Lum et al., 2006). A lipid-shell-based and BCNU-loaded MB could carry drugs, thus protecting the BCNU from rapid degradation, and could also be activated by FUS to simultaneously achieve BBB opening and trigger the local release of BCNU (Ting et al., 2012).

Progress has also been made in the manufacturing of smart MBs equipped with multi-functions. An example is VEGF-ligand conjugated and BCNU encapsulated MBs which was designed to ensure targeted delivery to areas where the tumor vasculature shows signs of angiogenesis, characterized by overexpression of the VEGF-R2 receptor (Fan et al., 2013b). A DOX-loaded and SPIO-nanoparticle conjugated phospholipid-based MB structure (DOX-SPIO-MB) simultaneously produced BBB opening and drug delivery, while also serving as a dual contrast agent in both ultrasound and MR imaging to confirm drug quantification and deposition (Fan et al., 2013a). In addition, applying an external magnetic force to magnetic nanoparticles offers the possibility of active magnetic targeting (MT) of particular brain regions. We had previously used FUS-BBB disruption to improve the delivery of magnetic nanoparticles (MNPs) into the brains of small animals (Chen et al., 2010; Liu et al., 2010b). Using external magnetic targeting, highly magnetized MNPs followed a time-dependent deposition pattern in the sonicated brain, with concentrations increased up to 20-fold compared with the contralateral brain.

### CNS Gene Delivery

#### Gene Delivery Into CNS Using FUS-BBB Opening

Gene therapy has the benefit of long-term expression of a therapeutic protein with limited distribution and may potentially provide a better solution for neurodegenerative diseases. Attempts have been recently made to use focused ultrasound for gene delivery either through viral- or non-viral-type vectors for gene transport.

#### Viral-Vector Gene Delivery

Adeno-associated virus (AAV) is widely used to express and secrete encoded human genes through genetically engineered modification. AAV vectors for the treatment of CNS diseases rely on localized, direct injection into the brain (Ridet et al., 2000; Miranpuri et al., 2012), but the region of recombinant gene expression is highly restricted by the blood-brain barrier (BBB).

Thevenot et al. applied focused-ultrasound exposure with a self-complementary adeno-associated virus serotypes 9 (scAAV-9) carrying a green fluorescent protein (GFP) gene in mice brains (Thevenot et al., 2012). Hsu et al. used GFP-encoded recombinant adeno-associated virus serotype 2 (rAAV-2) as the viral vector; fluorescent microscopic quantitative analysis indicated a high degree of GFP expression in the ultrasound exposure areas (Hsu et al., 2013). Additional comparison with a direct local virus injection showed the expression level of GFP fluorescence via focused ultrasound was almost equivalent to that of direct gene injection (Hsu et al., 2013). Wang et al. also compared transfection efficiency with reporter genes encoded in rAAV-1 and rAAV-2 and combined with ultrasound facilitated BBB opening (Wang et al., 2015).

#### Non-viral-vector Gene Delivery for PD Treatment

Viral-vector based ultrasound-facilitated CNS gene delivery has shown potential for promoting long-term endogenous expression of neurotrophic factors in the brain. It can also significantly enhance the length of the effective therapeutic periodic window. However, viral-vectors change the administration route from local injection to intravenous systemic circulation, which could result in systemic immunogenicity (Yoon et al., 2014).

Naked plasmid DNA delivery without using viral vectors has been attempted. Rather than microbubbles, Negishi et al. developed a nanobubble system (using nanobubbles about 200 nanometers (nm) in diameter) that successfully induced BBB-opening (Negishi et al., 2015). We offered a similar strategy to evaluate naked plasmid DNA delivery and gene expression via ultrasound-facilitated BBB opening (Fan et al., 2016). The results suggest successful plasmid delivery and gene expression, but the expression level did not outperform the traditional direct viral-gene vector injection approach. On the other hand, liposome as a vector can be used to encapsulate plasmid DNA to protect the plasmid from being degraded and neutralized during circulation. Unlike a viral-vector which delays expression by at least 7 days post sonication, a non-viral gene approach showed a delay expression about 48 h after sonication (Fan et al., 2016).

Parkinson's disease (PD) is a progressive neurodegenerative disease result from loss of dopaminergic neurons in the substantia nigra pars compacta. Currently, the most commonly used therapeutic strategy for PD, a systemic dopamine replacement therapy, can only improve the clinical motor symptoms for various period of time (Shao and Le, 2019). We previously demonstrated the feasibility of synthesizing liposome-based gene vectors for CNS gene delivery to treat neurodegenerative disease on PD animal models (Lin et al., 2015, 2016). A recovery of dopamine and their key metabolite levels as well as a recovery of motor symptoms in PD animals indicated the promise of the liposome-MB system as a vector to facilitate gene delivery in the CNS. We also developed a novel cationic MB system for plasmid DNA loading. Due to the positive charge of the cationic MB surface, the negatively-charged plasmid can easily be conjugated on the lipid surface with high DNA payload yields via charge interaction (Fan et al., 2016).

### BBB Opening for AD Treatment

Focal CNS diseases with unsatisfied treatment results exhibit apparent therapeutic targets to be aimed at, such as malignant glioma and PD. However, the scenario is different in treating a diffuse CNS disease. Alzheimer's disease (AD) is a diffuse neurodegenerative disease result from the abnormal accumulation of amyloid beta (Aβ) plaques and is the most common cause of age-related dementia (Madav et al., 2019). Several therapeutic agents including monoclonal antibodies (mAbs), stem cells and genes are under development and in clinical trials, but a BBB-penetrating issue has limited the therapeutic effect of these large molecular agents. In contrast to focally enhanced drug or gene delivery by FUS system, two hurdles including a diffuse deposition of Aβ plaques and currently no effective drugs targeting on the root cause of AD limit the therapeutic potential on AD using FUS-BBB system. However, FUS-BBB opening has not only physical effects of loosening the cellular tight junctions but also of inducing neuromodulation and immunogenic responses (see section BBB opening for CNS immune-modulation). The multidirectional responses from different components (i.e., microglial activation) of the therapeutic area offer an opportunity to change the microenvironment and immunogenicity, which might be beneficial for disease control (Leinenga and Gotz, 2015).

Burgess et al. attempted to open the BBB at the bilateral hippocampus (with a total of 3 exposures at 7 day intervals) (Burgess et al., 2014). Aβ plaques were observed by 3 months in the animal model (TgCRND8) and a near 20% plaque reduction was observed. Behavioral tests also showed that memory function and cognitive performance can be significantly restored in AD animal models (Burgess et al., 2014). Leinenga et al. conducted a more frequent exposure in the whole animal brain (with a total of 7 daily exposures). In their results, the amyloid plaque was reduced by up to 75% with a clear improvement in behavioral tests (Leinenga and Gotz, 2015). Two potential mechanisms have been proposed for FUS-mediated plaque reduction in the AD model. First, FUS-induced BBB opening delivered the endogenous IgG and IgM from the periphery into the brain, contributing to plaque clearance. Second, mild immune responses are induced by FUS and microglia was activated to internalize amyloid, contributing to plaque reduction (Jordao et al., 2013; Leinenga and Gotz, 2015).

We sought to determine if the use of FUS exposure to enhance GSK-3 inhibitor (AR-A014418) delivery can trigger the down regulation of Aβ synthesis and overexpression (Hsu et al., 2018). Microglia/immunogenic activation caused by FUS-BBB opening alone has been shown to be useful in removing existing plaque, thus adding GSK-3 inhibitor to decrease plaque synthesis presents a supplementary strategy to further reduce plaque deposition. An IHC examination showed GSK-3 inhibitor effectively reduced GSK-3 activity by up to 61.3%. FUS-induced BBB opening combined with GSK-3 inhibitor delivery had an additive effect on plaque reduction efficiency (39.6%, compared to 15.1% with FUS-BBB opening alone and 22.6% with GSK-3 inhibitor administration alone) (Hsu et al., 2018).

### BBB Opening for CNS Immune-Modulation

Focused ultrasound pulsation with microbubbles has been shown to trigger local immune response for tumor suppression (Liu et al., 2012). The discovery of the meningeal lymphatic system within the CNS helps explain the therapeutic role of systemic immune cells in various brain disorders. FUS-BBB opening could enhance delivery of immune-stimulating agents such as interleukin-12 (IL-12) (Chen et al., 2015a) or immune check point inhibitors such as anti-cytotoxic T-lymphocyte-associated antigen 4 (CTLA-4) monoclonal antibodies (mAb) to affect the tumor immunosuppressive microenvironment (Curley et al., 2017). Aside from delivering therapeutic agents to the brain, the procedure itself may exert some immune-related effects. For innate immune response, concentrations of several proinflammatory cytokines and heat shock proteins have been found to be transiently increased within 24 h following FUS exposure. FUS was also found to activate microglia, astrocytes, macrophages, and NK cells, and to enhance the infiltration capabilities of dendritic cells (DCs) as well as other antigen presenting cells in the treated tumor (Cohen-Inbar et al., 2016). For adaptive immunity, MB-assisted pulsed FUS stimulation was found to enrich cytotoxic T lymphocyte (CTL) infiltration, increase the CTL-to-regulatory T cell ratio and retard tumor growth in a murine model (Chen et al., 2015b). FUS-induced BBB opening results in CNS immune modulation in the following ways. First, it increases local BBB permeability to allow penetration of circulating mAbs or cytokines for immune-regulation. It also recruits or adjusts desirable immune cells to infiltrate and target the lesion. Finally, it activates neuroglial cells and other innate cells to create microenvironment conditions unfavorable to the disease. These three mechanisms suggest future applications for FUS-induced BBB opening for neuro-immune modulation and immunotherapy.

## Clinical Proof-of-Concept Testing

### Focused Ultrasound Device Design for Clinical Use

Recent advances in ultrasound-induced BBB opening techniques have led to the development of translational work on human patients. A recent study demonstrated the safety of an MRI-guided FUS platform for BBB opening in patients with brain tumors and AD (Dasgupta et al., 2016; Lipsman et al., 2018). Once the target has been confirmed to preoperatively assist procedure guidance, the focal energy deposition can be identified through a slight temperature rise due to weak FUS energy exposure, but exact occurrence of BBB opening can only be confirmed postoperatively via contrast-enhanced MRI via Gd-DTPA.

In addition, a planar implantable ultrasound device has also been developed using a surgical burr hole as an insertion point for an ultrasound disk to sonicate brain regions without the need for additional guidance procedures (SonoCloud®, CarThera) (Carpentier et al., 2016).

Neuronavigation systems have also been designed to guide FUS for precise BBB opening (Wei et al., 2013b; Wu et al., 2018). Preoperative diagnostic scans (CT or MRI) are first analyzed, followed by a registration process that allows for the 3D localization of the surgical tools to assist neurosurgeons in mapping the safest, least invasive path to the target site. Neuronavigation-guided FUS brain drug delivery has been shown to be feasible, with precision comparable to neurosurgical stereotactic procedures (Wei et al., 2013a; Wu et al., 2018).

### Brain Tumor Trial

The major hurdle to treating brain tumors is the delivery of drugs including chemotherapeutic and targeted therapy agents. One currently emerging concept focuses on turning the immune-suppressive environment, or “cold tumor,” into an immune-activated “hot” tumor. This approach has been shown to be effective in terms of improving therapeutic agent delivery and immune-modulation effect using the FUS-BBB opening technique. Based on significant preclinical evidence, clinical trials of FUS-BBB opening via various devices have been conducted since 2014 (Table 1). A total of six trials have been conducted on glioblastoma patients using a variety of devices including SonoCloud® (CarThera), ExAblate® (InSightec), and NaviFUS® (NaviFUS cooperation), both with and without chemotherapy regimens, such as carboplatin, doxorubicin and temozolomide. One trial in Spain focuses on patients with breast cancer brain metastases. All announced trials are still recruiting participants, with the exception of one trial using SonoCloud® in treating recurrent glioblastoma patients. A repeated opening of the BBB using implanted pulsed ultrasound device (SonoCloud®), in combination with Sonovue® (dose: 0.1 ml/kg) at an acoustic pressure ranged from 0.5 to 1.1 MPa, has been shown to be safe and well tolerated in treating recurrent GBM patients (Carpentier et al., 2016).

**Table 1:** Summary of the FUS-BBB opening Clinical Trials.

	**Trial no**.	**Study start day**	**Study title**	**Indication**	**Intervention and parameters**	**Location**	**Status**
**BRAIN TUMOR**							
1	NCT02253212	2014/7	Safety of BBB Opening with the SonoCloud (SONOCLOUD)^a^	Recurrent glioblastoma	SonoCloud; *n =* 27 0.5–1.1MPa Microbubble: Sonovue® (0.1 ml/kg) Drug: Carboplatin	France	Completed
2	NCT02343991	2014/10	BBB Disruption Using Transcranial MRI-Guided Focused Ultrasound^b^	Brain Tumor	Exablate; *n =* 10 PCD-based power regulation Microbubble: Definity® (4 μ/kg) Drug: Doxorubicin	Canada	Active, not recruiting
3	NCT03626896	2018/8/17	Safety of BBB disruption using NaviFUS system in recurrent glioblastoma multiforme (GBM) patients^c^	Recurrent glioblastoma	NaviFUS; *n =* 6 Escalated exposure average 10–16W; Microbubble: Sonovue® (0.1 ml/kg)	Taiwan	Recruiting
4	NCT03712293	2018/8/28	ExAblate Blood-Brain Barrier Disruption for Glioblastoma in Patients Undergoing Standard Chemotherapy^d^	Glioblastoma patients undergo adjuvant Temozolomide	Exablate; *n =* 10 PCD-based power regulation Microbubble: Definity® (4 μl/kg) Drug: Temozolomide	Korea	Recruiting
5	NCT03616860	2018/10	Assessment of Safety and Feasibility of ExAblate Blood-Brain Barrier (BBB) Disruption for Treatment of Glioma^e^	Glioblastoma	Exablate; *n =* 20 PCD-based power regulation Microbubble: Definity® (4 μl/kg) Drug: Lipodox, Temozolomide	Canada	Recruiting
6	NCT03551249	2018/11	Assessment of Safety and Feasibility of ExAblate Blood-Brain Barrier (BBB) Disruption^f^	Glioblastoma	Exablate; *n =* 20 Power level: PCD-based power regulation Microbubble: Definity® (4 μl/kg) Drug: Temozolomide	USA	Not yet recruiting
7	NCT03714243	2018/12	Blood Brain Barrier Disruption (BBBD) Using MRgFUS in the Treatment of Her2-positive Breast Cancer Brain Metastases^g^	Breast cancer with brain metastases	Exablate; *n =* 10 Power level: PCD-based power regulation Microbubble: Definity® (4 μl/kg)	N/A	Not yet recruiting
**ALZHEIMER'S DISEASE**							
8	NCT02986932	2016/12	BBB Opening Using Focused Ultrasound with IV Contrast Agents in Patients with Early Alzheimer's Disease^h^	Alzheimer's Disease	Exablate; *n =* 6 Power level: PCD-based exposure level regulation (average 4.6W) Microbubble: Definity® MB (4μl/kg)	Canada	Completed
9	NCT03119961	2017/6/26	Blood Brain Barrier Opening in Alzheimer's Disease (BOREAL1)^i^	Alzheimer's Disease	Sonocloud; *n =* 10 Power level: 0.5–1.1MPa, Microbubble: Sonovue® MB (0.1 ml/kg)	France	Recruiting
10	NCT03671889	2018/9/28	ExAblate Blood-Brain Barrier (BBB) Disruption for the Treatment of Alzheimer's Disease^j^	Alzheimer's Disease	Exablate; *n =* 10 Power level: PCD-based exposure level regulation Definity® MB (4μl/kg)	USA	Recruiting
11	NCT03739905	2018/12	ExAblate Blood-Brain Barrier Opening for Treatment of Alzheimer's Disease^k^	Alzheimer's Disease	Exablate; *n =* 30 PCD-based exposure level regulation Microbubble: Definity® MB (4μl/kg)	Canada	Not yet recruiting
**OTHERS**							
12	NCT03321487	2018/4/13	BBB Opening Using MR-Guided Focused Ultrasound in Patients with Amyotrophic Lateral Sclerosis^l^	Amyotrophic Lateral Sclerosis	Exablate; *n =* 8 PCD-based exposure level regulation Microbubble: Definity® (4μl/kg)	Canada	Recruiting
13	NCT03608553	2018/12	Evaluate Temporary Blood Brain Barrier Disruption in Patients with Parkinson's Disease Dementia^m^	Parkinson's Disease Dementia	Exablate; *n =* 10 PCD-based exposure level regulation Microbubble: Definity® (4μl/kg)	Spain	Recruiting

a *ClinicalTrials.gov. Ahmed Idbaih (MD): Groupe Hospitalier Pitié Salpetriere (France) (2014). Identifier NCT02253212, Safety of BBB Opening With the SonoCloud (SONOCLOUD). Available online at: http://clinicaltrials.gov/ct/show/NCT02253212 (Accessed January 15, 2019)*.

b *ClinicalTrials.gov. InSightec: Sunnybrook Health Science Center (Canada) (2014). Identifier NCT02343991, BBB Disruption Using Transcranial MRI-Guided Focused Ultrasound. Available online at: http://clinicaltrials.gov/ct/show/NCT02343991 (Accessed January 15, 2019)*.

c *ClinicalTrials.gov. Kuo-Chen Wei (MD): Linkou Chang Gung Memorial Hospital (Taiwan). (2018). Identifier NCT03626896, Safety of BBB disruption using NaviFUS system in recurrent glioblastoma multiforme (GBM) patients. Available online at: http://clinicaltrials.gov/ct/show/NCT03626896 (Accessed January 15, 2019)*.

d *ClinicalTrials.gov. Martine Bernstein: Severance Hospital, Yonsei University Health System (Korea). (2018). Identifier NCT03712293, ExAblate Blood-Brain Barrier Disruption for Glioblastoma in Patients Undergoing Standard Chemotherapy. Available online at: http://clinicaltrials.gov/ct/show/NCT03712293 (Accessed January 15, 2019)*.

e *ClinicalTrials.gov. Nir Lipsman (MD): Sunnybrook Health Science Center (Canada). (2018). Identifier NCT03616860, Assessment of Safety and Feasibility of ExAblate Blood-Brain Barrier (BBB) Disruption for Treatment of Glioma. Available online at: http://clinicaltrials.gov/ct/show/NCT03616860 (Accessed January 15, 2019)*.

f *ClinicalTrials.gov. InSightec. (2019). Identifier NCT03551249, Assessment of Safety and Feasibility of ExAblate Blood-Brain Barrier (BBB) Disruption. Available from: http://clinicaltrials.gov/ct/show/NCT03551249 (Accessed January 15, 2019)*.

g *ClinicalTrials.gov. Nir Lipsman (MD): Sunnybrook Health Science Center (Canada). (2018). Identifier NCT03714243, Blood Brain Barrier Disruption (BBBD) Using MRgFUS in the Treatment of Her2-positive Breast Cancer Brain Metastases. Available online at: http://clinicaltrials.gov/ct/show/NCT03714243 (Accessed January 15, 2019)*.

h *ClinicalTrials.gov. Nir Lipsman (MD): Sunnybrook Health Science Center (Canada). (2016). Identifier NCT02986932, BBB Opening Using Focused Ultrasound with IV Contrast Agents in Patients with Early Alzheimer's Disease. Available online at: http://clinicaltrials.gov/ct/show/NCT02986932 (Accessed January 15, 2019)*.

i *ClinicalTrials.gov. Stephane Epelbaum(MD): APHP - Pitié-Salpêtrière Hospital (France). (2017). Identifier NCT03119961, Blood Brain Barrier Opening in Alzheimer's Disease (BOREAL1). Available online at: http://clinicaltrials.gov/ct/show/NCT03119961 (Accessed January 15, 2019)*.

j *ClinicalTrials.gov. InSightec: Weill Corneal Medicine (US); Weill Corneal Medicine, The Ohio State University-Wexner Medical Center, West Virginia University Rockefeller Neuroscience Center (US). (2018). Identifier NCT03671889, ExAblate Blood-Brain Barrier (BBB) Disruption for the Treatment of Alzheimer's Disease. Available online at: http://clinicaltrials.gov/ct/show/NCT03671889 (Accessed January 15, 2019)*.

k *ClinicalTrials.gov. InSightec: Sunnybrook Health Science Center (Canada). (2018). Identifier NCT03739905, ExAblate Blood-Brain Barrier Opening for Treatment of Alzheimer's Disease. Available online at: http://clinicaltrials.gov/ct/show/NCT03739905 (Accessed January 15, 2019)*.

l *ClinicalTrials.gov. InSightec: Sunnybrook Health Science Center (Canada). (2018). Identifier NCT03321487, BBB Opening Using MR-Guided Focused Ultrasound in Patients with Amyotrophic Lateral Sclerosis. Available online at: http://clinicaltrials.gov/ct/show/NCT03321487 (Accessed January 15, 2019)*.

m *ClinicalTrials.gov. Jose Obeso: HM Hospitales Puerta del Sur – CINAC (Spain). (2018). Identifier NCT03608553, Evaluate Temporary Blood Brain Barrier Disruption in Patients with Parkinson's Disease Dementia. Available online at: http://clinicaltrials.gov/ct/show/NCT03608553 (Accessed January 15, 2019)*.

*BBB, blood brain barrier; FUS, focused ultrasound; MB, microbubble; PCD, passive cavitation detection*.

### AD Trial

For Alzheimer disease, exciting results from animal experiments have shown a possibility for decreasing Aβ deposits via scanning ultrasound with BBB opening parameters (Burgess et al., 2014; Leinenga and Gotz, 2015). Since 2016, four clinical trials including SonoCloud® (CarThera) and ExAblate® (InSightec) have been applied on early AD patients to evaluate safety and feasibility. Lipsman et al. conducted a phase I trial on 5 AD patients using an PCD-feedback power regulation approach but with an average exposure level of 4.6W with Definity® (dose: 4 μl/kg), demonstrating a safe, reversible and repeated opening of BBB by MRgFUS device (ExAblate®) (Lipsman et al., 2018). Recently, a single-arm, non-randomized phase IIa trial has been announced in Canada to evaluate the efficacy of FUS-BBB opening on treating AD patients (trial number: NCT03739905, Table 1).

### ALS and PD Dementia Trial

Phase I trials are currently ongoing for amyotrophic lateral sclerosis (ALS) and Parkinson's disease dementia using an MRgFUS device (ExAblate®) for BBB opening (Trial numbers NCT03321487 and NCT03608553; Table 1).

### Technical Gap of Translational Application From Preclinical to Clinical

Although substantially accumulative proof-of-concept preclinical studies have concluded and demonstrated the potential benefit of utilizing FUS-BBB opening technique for CNS disease treatments, a number of technical gaps need to be filled prior to its wide clinical translation. First, the heterogeneity of intracranial structures such as gray and white matter and dense vasculature, as well as the thick and uneven skull, may cause substantial FUS beam distortion and transcranial pressure attenuation when ultrasound passes through the skull. Moreover, concerns about physical parameters and individual BBB-opened threshold level variation due to different treatment portion containing various vascular density or personalized variation are critical issues that need to addressed. In addition, current on-going clinical trials employed various medical devices combined with various types of microbubbles bring extra difficulty to unify the ultrasound dose to be delivered into patient brain. Last but not least, the current standard to verify the occurrence of BBB opening can only be confirmed via post-operative MRI contrast agent administration and the process so far lacks tools for intraoperative BBB-opened monitoring.

For filling the above technical gaps, a promising scheme is to utilize the PCD as an tool to (1) provide correlations between delivery efficiency and BBB opening volume in steering the treatment as real-time monitoring, and (2) to provide real-time means to control the occurrence of BBB opening to avoid adverse effect (Wu et al., 2016, 2018; Xia et al., 2016; Liu et al., 2018). In addition, a personalized treatment planning tools need to be developed to individually determining FUS physical parameters with dedicated consideration of transcranial focal beam distortion and compensation prior to the treatment.

For now, three different types of therapeutic ultrasound devices, including the implanted ultrasound device (SonoCloud®), the extracorporeal fixed stereotactic frame-based MRI-guided device (Exablate®) to the frameless neuronavigation-guided device (NaviFUS®) are available on the market to treat and to explore the efficacy in human clinical trials. A trend toward a less invasive FUS modality with patient-centered protocol design, in a meanwhile, maintaining the treatment efficacy with proper parameters under on-line feedback would be the paramount goal in FUS-BBB development.

## Conclusion

The use of focused ultrasound for blood-brain barrier opening is an innovative and non-invasive means to achieve drug delivery deep within the CNS along with other therapy modalities. Compared to other drug delivery approaches, focused ultrasound BBB opening provides significant advantages in terms of locality, non-invasiveness, and effect reversibility. Over the past decade, significant advances have been made in technological development and preclinical validation, and this technique is now entering clinical trials for patients suffering from brain tumors, Alzheimer's disease, ALS, and Parkinson's disease dementia. Preliminary results have confirmed safety and efficacy for brain tumor treatment, and show significant promise for additional indications, raising the potential for focused ultrasound blood-brain barrier opening to emerge as an important tool for CNS disease treatment.

## Author Contributions

K-TC, K-CW, and H-LL together drafted, organized, and finalized the manuscript

### Conflict of Interest Statement

H-LL serves as the technical consultant of NaviFUS Inc. Taiwan. The remaining authors declare that the research was conducted in the absence of any commercial or financial relationships that could be construed as a potential conflict of interest.
